# Supplementary material for: Process Development for GMP-Grade Full Extract Cannabis Oil: Towards Standardized Medicinal Use
Source: Pharmaceutics. 2025 Jun 28;17(7):848. doi: 10.3390/pharmaceutics17070848 (PMC12300620; doi:10.3390/pharmaceutics17070848)
Supplement: Supplementary file 1 [file pharmaceutics-17-00848-s001.zip › pharmaceutics-3669469-supplementary.pdf]

pharmaceutics

MDPI

Article

Process Development for GMP-Grade Full-Spectrum Cannabis Extract Oil: Towards Standardized Medicinal Use

Maria do Céu Costa[1,2], Ana Patrícia Gomes[1,3], Iva Vinhas[3], Joana Rosa[3], Filipe Pereira[3], Sara Moniz[3], Elsa M Gonçalves[4,5], Miguel Pestana[4], Mafalda Silva[4,6], Luís Monteiro Rodrigues[1], Anthony DeMeo[3], Logan Marynissen[3], António Marques da Costa[3], Patrícia Rijo[1], and Michael Sassano[3]

[1] CBIOS - Universidade Lusófona's Research Center for Biosciences & Health Technologies, Campo Grande 376, 1749-024 Lisboa, Portugal; maria.costa@ulusofona.pt (M.d.C.C.); pg@somaipharma.eu (A.P.G.); monteiro.rodrigues@ulusofona.pt (L.M.R.)
[2] NICiTeS, Polytechnic Institute of Lusophony, ERISA-Escola Superior de Saúde Ribeiro Sanches, Rua do Telhal aos Olivais 8, 1950-396 Lisboa, Portugal.
[3] SOMAÍ Pharmaceuticals, R. 13 de Maio 52, 2580-507, Carregado, Portugal; imv@somaipharma.eu (I.V.); jr@somaipharma.eu (J.R.); fjp@somaipharma.eu (F.P.); sjm@somaipharma.eu (S.M.); symbioticad@gmail.com (A.D.M.); lm@somaipharma.eu (L.M.); amc@somaipharma.eu (A.M.-d-C.); mailto.ms@somaipharma.eu (M.S.)
[4] INIAV, Instituto Nacional de Investigação Agrária e Veterinária I.P., Quinta do Marquês, 2780-157 Oeiras, Portugal; elsa.goncalves@iniav.pt (E.M.G.); miguel.pestana@iniav.pt (M.P.); mafaldasdos@gmail.com (M.Si.)
[5] GeoBiotec—GeoBioTec Research Institute, Faculdade de Ciências e Tecnologia, Universidade Nova de Lisboa, Campus da Caparica, 2829-516 Caparica, Portugal
[6] Faculdade de Ciências e Tecnologia, Universidade Nova de Lisboa, Campus da Caparica, 2829-516 Caparica, Portugal;
* Correspondence: patricia.rijo@ulusofona.pt (P.R.); maria.costa@ulusofona.pt (M.d.C.C.)

Supplementary Materials

Table S1. Content of phenolics, flavonoids, chlorophyll, and waxes (% w/w) in Flower, SOMAÍ FECO, and Purified SOMAÍ FECO, highlighting the impact of extraction and purification on non-cannabinoid constituents.

Content (% w/w)	Flower (dry base)	SOMAÍ FECO	Purified SOMAÍ FECO
**Phenolics**	0.14±1	1.4±0.1	1.4±0.1
**Flavonoids**	0.12±1	1.0±0.2	1.1±0.3
**Chlorophyll**	3±1	0.11±0.04 x 10^{-3}	0.03±0.02x 10^{-3}
**Waxes**	9.2 ± 3.1	4.1±1.2 x 10^{-3}	3.8 ± 0.4 x 10^{-3}

Table S2. Quantification of terpenes (% w/w) in the high-THC flower, crude high-THC FECO extract (SOMAÍ FECO), purified high-THC FECO extract (Purified SOMAÍ FECO), and two final CBD/THC (10:10) oral solutions (OS) obtained by adding terpenes in controlled reintroduction.

Terpenes	Flower	SOMAÍ FECO	Purified SOMAÍ FECO	OS1	OS2
alpha-pinene	0.0241	0.0019	ND	0.0685	0.03814
camphene	0.0066	0.0009	ND	0.00644	0.00894
beta-pinene	0.0341	0.0049	0.00004	0.05754	0.06034
myrcene	0.1332	0.0085	0.0006	0.58527	0.13955
3-carene	0.0002	0.0003	0.0001	0.00123	0.00031
alpha-terpinene	0.0009	0.0007	ND	0.00175	0.00214
cymene	0.0002	ND	ND	0.00125	0.00114
D.L-limonene	0.1699	0.0294	0.0004	0.31692	0.48473
gamma-terpinene	0.0011	0.0005	0.0001	0.00182	0.00409
terpinolene	0.0045	0.0035	0.00001	0.03621	0.01637
L-fenchone	0.0048	0.0038	ND	ND	ND
linalool	0.0477	0.0576	0.0006	0.05076	0.10022
fenchol	0.0394	0.0603	0.0005	0.01658	0.02523
camphor	ND	ND	ND	0.00004	ND
iso borneol	0.0004	0.0009	0.00001	0.00046	0.00039
borneol	0.0124	0.0295	0.0004	0.00313	0.00428
D.L-menthol	ND	0.0003	ND	0.00016	0.00018
alpha-terpineol	0.0399	0.1047	0.0012	0.0092	0.01486
B-citronellol	0.0019	0.0043	0.0001	0.00044	0.00069
R-pulegone	ND	ND	ND	ND	ND
geraniol	0.0025	0.0104	0.0002	0.00062	0.00142
geranyl acetate	ND	0.0009	ND	ND	ND
alpha-cedrene	ND	ND	ND	0.00298	0.00936
beta-caryophellene	0.2336	0.3324	0.0032	0.15994	0.43231
alpha-humulene	0.0724	0.1169	0.0017	0.05095	0.1386
nerolidol	0.0302	0.116	0.0252	0.00396	0.00875
cedrol	ND	ND	ND	ND	ND
beta-eudesmol	0.0282	0.112	0.0557	0.00619	0.00514
alpha-bisabolol	0.0819	0.3026	0.1694	0.01868	0.01708
phytol	0.0206	0.0133	0.032	0.00491	0.00619
**Total**	**0.9907**	**1.3165**	**0.29146**	**1.40593**	**1.52045**

ND-not detected.

Table S3. Summary of the questions and results regarding the perception and acceptance of cannabis SOMAÍ solutions for sample A and B.

Question	Sample A	Sample B
Is the taste of the sample pleasant?	Yes – 9<br>No – 3	Yes – 8<br>No – 1<br>Indifferent – 3
What tastes can you identify in the extract?	Herbal – 11<br>Fruity – 1	Herbal – 10<br>Other – 2
Did you identify any taste that caused discomfort or immediate rejection?	Indifferent – 1<br>No – 11	No – 12
Does any taste linger for a long time after consumption?	Yes – 5<br>No – 7	No – 12
Is this taste pleasant or unpleasant?	Pleasant – 5<br>Indifferent – 7	Pleasant – 2<br>Indifferent – 10
If you had to consume the oil daily, would the taste of the oil influence your decision to continue the use?	Yes – 3<br>No – 9	Yes – 3<br>No – 9
Describe the acceptance of the sample	Very acceptable – 7<br>Indifferent – 2<br>Not acceptable – 3	Very acceptable – 10<br>Indifferent – 2

Table S4. Summary of the questions and results regarding the perception and acceptance of cannabis solutions for sample C and D.

Question	Sample C	Sample D
Is the taste of the sample pleasant?	Yes – 4<br>Indifferent – 2<br>No – 1	Yes – 1<br>Indifferent – 3<br>No – 3
What tastes can you identify in the extract?	Herbal – 6<br>Other – 1	Herbal – 4<br>Other – 2
Did you identify any taste that caused discomfort or immediate rejection?	No – 7	Yes – 2<br>Indifferent – 4<br>No – 1
Does any taste linger for a long time after consumption?	Yes – 3<br>No – 4	Yes – 3<br>No – 4
Is this taste pleasant or unpleasant?	Pleasant – 2<br>Indifferent – 4<br>Unpleasant - 1	Indifferent – 4<br>Unpleasant - 3
If you had to consume the oil daily, would the taste of the oil influence your decision to continue the use?	Yes – 1<br>Indifferent – 1<br>No – 5	Yes – 2<br>Indifferent – 1<br>No – 4
Describe the acceptance of the sample	Very acceptable – 5<br>Indifferent – 1<br>Not Acceptable – 1	Very acceptable – 2<br>Indifferent – 3<br>Not Acceptable – 1
